# Comprehensive Evaluation of Referral Practices From General Practitioners to Balbriggan CMHT, Dublin: Audit Cycle Overview

**DOI:** 10.1192/bjo.2025.10599

**Published:** 2025-06-20

**Authors:** Umer Saeed Haroon, Shahzaib Maqsood, Eimear Kelly

**Affiliations:** 1 Ashlin Centre, Dublin, Ireland; 2 RCSI, Dublin, Ireland

## Abstract

**Aims:** The aim of this audit was to evaluate and enhance referral practices from general practitioners (GPs) to the Balbriggan Community Mental Health Team (CMHT), Dublin. Appropriate referrals are crucial for effective mental health care. The initial audit, conducted in mid-2024, sought to identify barriers to successful referrals, particularly regarding psychotherapy initiation and medication management. Following these findings, guidelines were developed and disseminated to GPs to improve the referral process. A follow-up audit was then conducted to assess the impact of these interventions on referral practices and identify any remaining challenges.

**Methods:** An audit cycle was conducted, comprising a retrospective initial audit followed by a prospective follow-up audit. The initial audit reviewed 110 referrals from 1 May to 1 August 2024, while the follow-up audit analysed 77 referrals from 1 September to 30 November 2024. Following the initial audit, local guidelines were created and shared with GPs on 19 August 2024, focusing on appropriate referral procedures, psychotherapy initiation, and medication management. Data collection focused on referral acceptance rates, reasons for rejection, and the initiation of psychotherapy and psychotropic medication. The effectiveness of the guidelines was also evaluated.

**Results:** The results of the audits showed significant improvements in referral practices. In the initial audit, 110 referrals were reviewed, resulting in 60 accepted (54.5%) and 50 rejected (45.5%). Key barriers included 16% of patients not receiving psychotherapy and 11% receiving suboptimal medication dosages. Additionally, 9% of referrals were declined due to non-initiation of psychotropic medications, indicating GPs’ hesitancy to refer patients without prior treatment.

In contrast, the follow-up audit, which reviewed 77 referrals, showed a marked increase in acceptance rates, with 71 accepted (92.2%) and only 6 rejected (7.8%). However, 14% of patients still did not receive psychotherapy, suggesting persistent hesitancy among GPs. Notably, the percentage of rejected referrals for ADHD/ASD assessments increased from 21% to 33%, indicating that misalignment between GP expectations and CMHT services remains a challenge.

**Conclusion:** This audit demonstrates the importance of effective communication and collaboration between GPs and the CMHT in enhancing referral practices. The implementation of guidelines led to improved referral acceptance rates. However, challenges still exist regarding psychotherapy initiation and specific service offerings, particularly for ADHD/ASD assessments. Ongoing monitoring and education for GPs are essential to sustain these improvements and ensure optimal patient access to mental health care.

